# Supplementary figures and images for: Perception Toward Wolves Are Driven by Economic Status and Religion Across Their Distribution Range
Source: Animals (Basel). 2025 Apr 23;15(9):1196. doi: 10.3390/ani15091196 (PMC12071121; doi:10.3390/ani15091196)

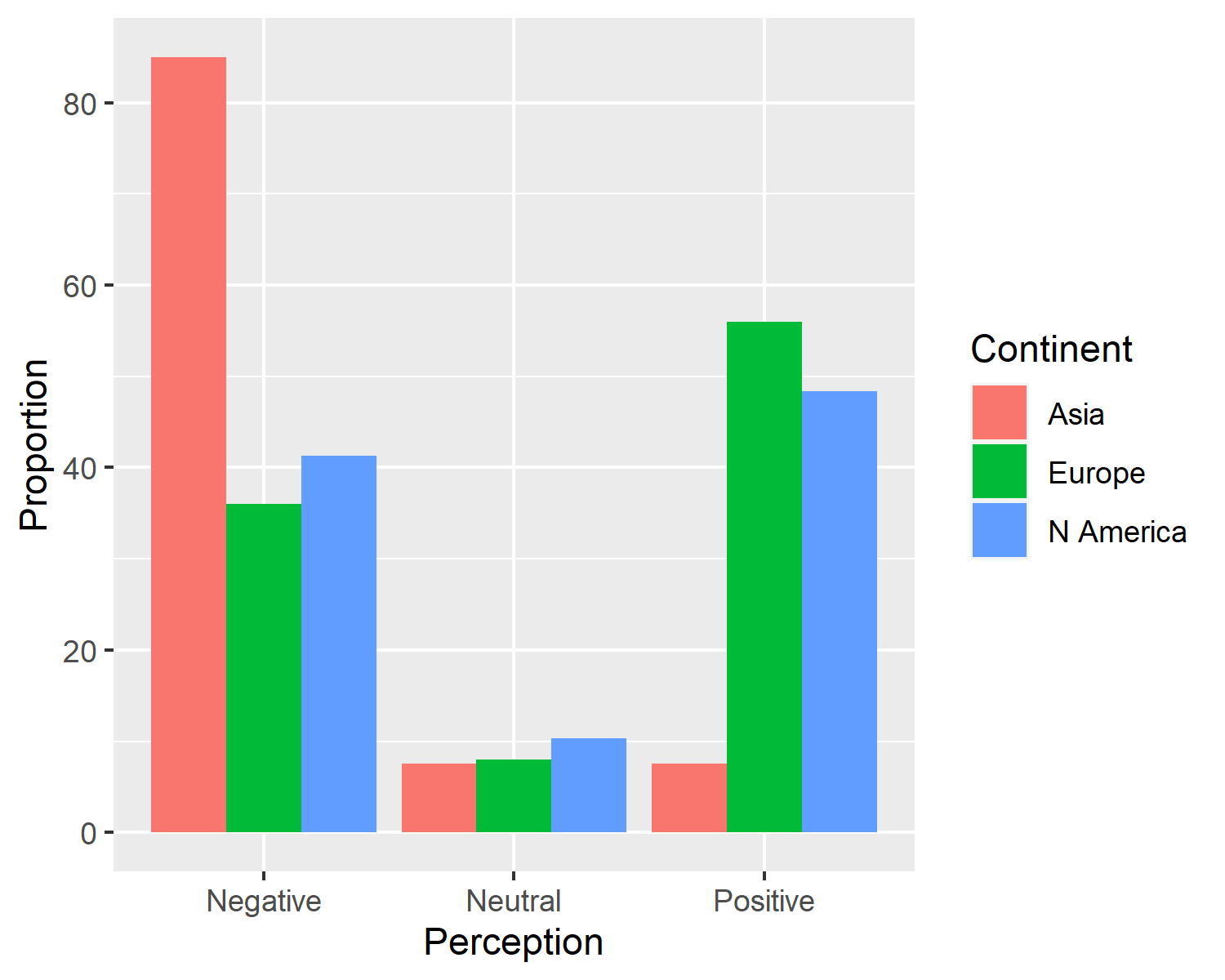

Supplement: Supplementary file 1 [file animals-15-01196-s001.zip › Figure 1_S1_New.tiff]

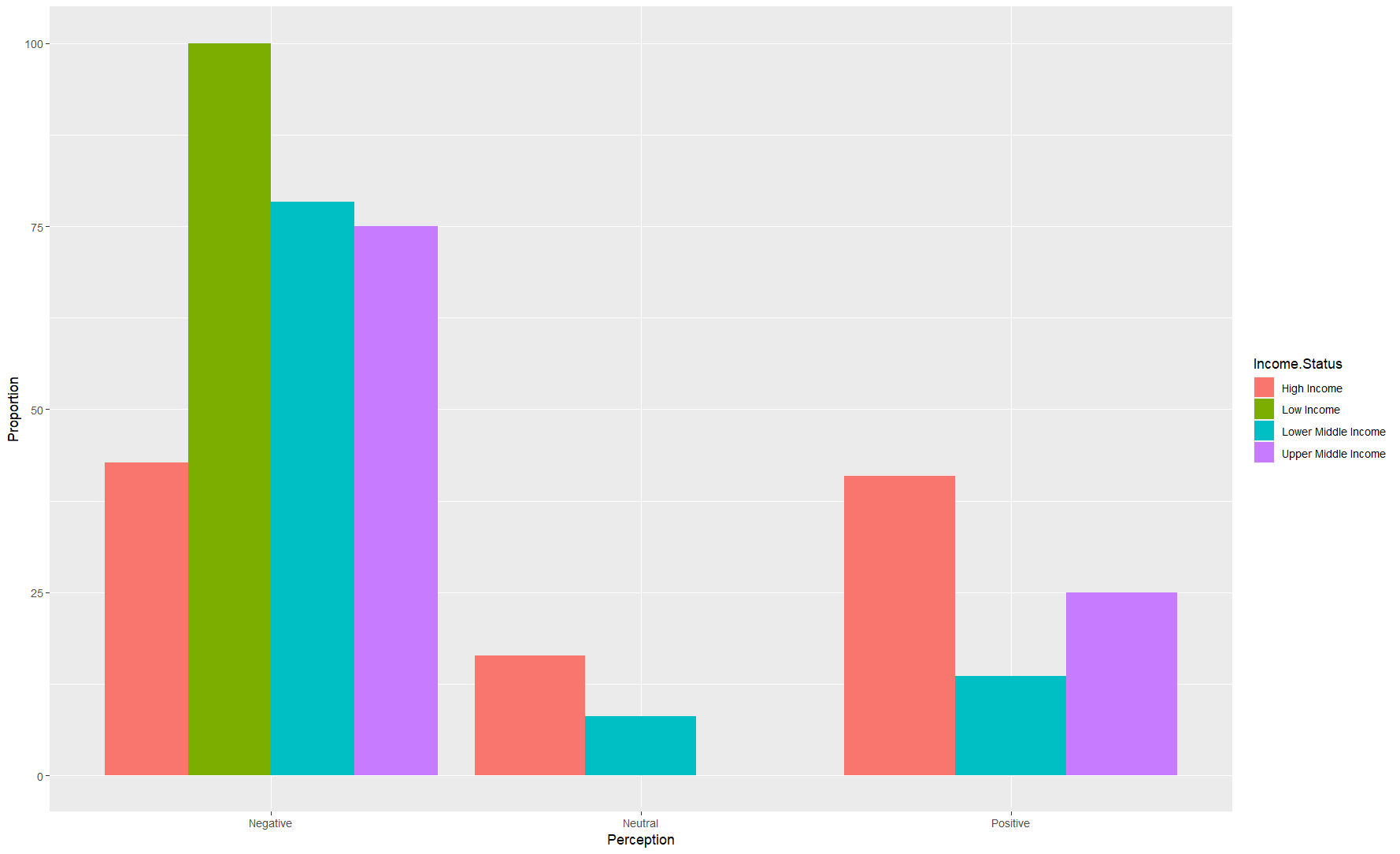

Supplement: Supplementary file 1 [file animals-15-01196-s001.zip › Figure 2_S2_New.tiff]
